# Inclusive universities: promoting gender equality and sex-gender diversity

**DOI:** 10.3389/fpubh.2026.1766440

**Published:** 2026-02-12

**Authors:** Begoña Sánchez Torrejón, Macarena Machín Álvarez

**Affiliations:** Department of Didactic, Faculty of Education, University of Cádiz, Cádiz, Spain

**Keywords:** inclusive education, LGBTIQA+, university, sex-gender diversity, sexual health, gender equality, well-being

## Abstract

**Introduction:**

This study investigates the implementation of gender equality and sex-gender diversity policies in a higher education context, within the G-FORCE Project funded by Erasmus+.

**Methods:**

Two focus groups (*N* = 20) were conducted with participants from the academic community, administrative staff, research personnel, and external stakeholders, ensuring diverse generational and gender representation. Data were collected through guided focus group discussions, transcribed, and analyzed using reflexive thematic analysis with an interpretive approach.

**Results:**

Three dimensions emerged: (1) knowledge and awareness of gender equality and sex-gender diversity, (2) institutional practices and university policies, and (3) university culture. Results indicate that although participants are aware of existing protocols and training, these initiatives are perceived as isolated, minimally innovative, and largely symbolic.

**Conclusion:**

A gap exists between formal policies and actual practices, reflecting a bureaucratic culture that limits inclusive transformation. Resistance is present among faculty and students committed to equality, yet systemic barriers persist. The study concludes that effective inclusion requires structural change, curricular integration of inclusive perspectives, creation of safe spaces, participatory governance, and alignment of institutional culture with gender equality and sex-gender diversity.

## 1 Introduction

The university have a fundamental responsibility to promote, protect, and uphold the rights of LGBTIQA+ people (lesbian, gay, bisexual, trans, intersex, queer, and other gender identities and sexual orientations) at the educational, social, welfare, and health levels but can be also actors that reproduce the social and cultural prejudices and stigmas latent in society.

The key is that universities not only train future professionals in various disciplines, but also serve as spaces where human rights, inclusion and equal opportunities for LGBTIQA+ people must be put into practice and gender equality must be promoted (1, 2). In this regard, it is essential for universities to adopt clear policies that protect LGBTIQA+ individuals from any form of discrimination, whether based on sexual orientation, gender identity, or gender expression. The university— a crucible of knowledge, innovation, and critical thinking—stands as a fundamental institution in shaping just and equitable societies (3). Universities must be places where LGBTIQA+ individuals can live and express themselves without fear of stigma, where they can freely develop their diverse expressions, identities, and sexual orientations—spaces of well-being (4).

As Paredes Fuentes (5) notes, we cannot forget that the university is not merely a space where knowledge is transmitted; it is a living and dynamic institution with the power and responsibility to drive social change in the area of gender equality and LGBTIQA+ rights. Through teaching committed to LGBTIQA+ rights, research grounded in a gender perspective, and active university outreach, the university becomes a key pillar in building more just, equitable, and inclusive societies that fully guarantee the well-being of non-cisheteronormative students (6). The university’s commitment to gender equality and sex-gender diversity must be transversal, permeating its internal structure and functioning, and ensuring the comprehensive well-being and health of LGBTIQA+ individuals.

In light of the above, higher education must promote diversity and a culture of respect and equality for LGBTIQA+ people, fostering spaces for personal development and well-being within the university environment and helping eliminate all forms of LGBTIQA+ phobia. The Comisión Europea (7) promotes the idea that higher education must play its role in addressing social and democratic challenges, which means ensuring that the university is inclusive so that learning communities are civically aware and connected to their broader communities.

Spain’s Organic Law 2/2023 (8) on the University System establishes the obligation of universities to promote equality and non-discrimination— including on the grounds of sexual orientation, gender identity, or gender expression— and to adopt specific measures to safeguard the rights of LGTBIQ+ people.

- Article 8.1.b states that one of the functions of universities is to contribute to the social, economic, and cultural development of their environment, promoting gender equality and the social inclusion of all individuals, especially those who may be subject to discrimination for any reason, including sexual orientation, gender identity or expression, or social, cultural, racial, ethnic, or religious origin.
- Article 12.2.g establishes that the university’s strategic plan must include specific actions regarding equality and non-discrimination, particularly in relation to gender equality and the protection of the rights of LGTBIQ+ people.
- Article 38.2.g indicates that one of the responsibilities of the social council is to ensure compliance with the principles of equality, non-discrimination, and human rights protection, including gender equality and the protection of LGBTIQA+ rights.

However, the reality of LGBTIQA+ students in university environments still presents significant shortcomings, as they continue to experience LGBTIQA+ phobia, which directly affects their health and well-being. This perspective departs from the premise that higher education is not neutral; rather, it systematically reproduces dominant categories of gender, sexuality, and embodiment. Authors such as Fernández Hawrylak et al. (40) document that 28% of LGBTIQA+ students in Spain report feeling unsafe in mixed classrooms. Dueñas et al. (9) research on the university experiences of the LGBTIQA+ community has shown that non-cisheteronormative students are more likely to experience discrimination, harassment, and exclusion, which significantly affects both their academic performance and psychosocial well-being, with direct consequences for their health.

Along these lines, authors such as Meyer (10) argue that LGBTIQA+ people experience minority stress derived from social stigmatization, which is exacerbated in contexts where discriminatory discourses or practices exist, such as universities. While universities may promote inclusion at a declarative level, they can become hostile environments when university communities reproduce stereotypes or erase non-cisheteronormative identities, negatively affecting students’ well-being. Research by Woodford and Kulick (11) found that LGBTIQA+ students report significantly higher levels of anxiety and isolation when they perceive negative attitudes or offensive language in the university classroom. Similarly, Rankin et al. (41), in one of the most comprehensive studies on university climate, conclude that more than 20% of LGBTIQA+ students have experienced harassment or discrimination at their universities, revealing a systemic issue.

In addition to these direct aggression, we can also find other kind of violence when, through institutional practices, we invisibilize or marginalize dissident sex-gender identities. Talburt (12) argues that university policies often focus on tolerance rather than on profound transformation of institutional culture, resulting in superficial inclusion. In this sense, although universities can be influential institutions in promoting gender diversity and inclusion, not only in the higher education context, but also in society at large, they remain gendered and male-dominated with often wide disparities at regional and national levels (13, 14).

Given this situation, the project in which this study is embedded aims to empower the higher education community to fight gender bias and respect sexual orientation, providing, firstly, specialized information on gender discrimination, violence and sexual harassment within participating institutions. So, fight against LGBTIQA+ phobia in universities, as a type of gender-based violence, is not only a commitment to human rights, but an essential condition for building a democratic and plural academic community in which LGBTIQA+ individuals can find a safe and supportive environment across all domains (15). Through inclusive policies, education and awareness-raising, the creation of safe spaces, and active support for the community, the university can be a key actor in building a more just, equitable, and sex-gender diverse society. This situation underscores the urgent need for the G-FORCE project, which seeks to promote gender equality and respect for sexual orientation in universities by raising awareness and using innovative digital tools to strengthen the capacity of faculty and students in matters of gender equality and sex-gender diversity, ultimately improving the inclusion and well-being of LGBTIQA+ students.

## 2 Methodology

This study was conducted within the framework of the international higher education collaboration project ERASMUS+ titled *GFORCE: EnForcing Gender Equality and Contributing to Sexual Orientation Respect in Higher Education Institutions Alliance* (KA220-HED-0449BDD3). One of its work packages focuses on examining the status of partner universities regarding the promotion of gender equality and sex-gender diversity. To achieve this objective, a qualitative research design grounded in the interpretive paradigm was developed (16), based on the analysis of the perspectives and meanings expressed by key informants connected to the phenomenon under study (17).

To gain a deeper understanding of this reality, two focus groups were conducted. The first represented the internal university community—faculty, administrative and research staff, and students—while the second brought together actors representing the institution’s relationship with society as members of social organizations, public institutions, and research centres. Focus groups constitute a specific form of group interview in which discussion revolves around a defined topic and is guided by a facilitator (18). The purpose of the focus groups was to gather information on participants’ ideas, thoughts, attitudes, and perceptions regarding the university as an agent promoting gender equality and sex-gender diversity. In this sense, the data emerged from the interactions generated within the shared discussion space (19).

### 2.1 Participants

This study included *N* = 20 participants, distributed across two focus groups. Focus Group 1, labeled “educational community,” was composed primarily of students (*N* = 4), followed by teaching staff (*N* = 3), administrative staff (*N* = 2), and, finally, one representative from university research personnel (*N* = 1). Focus Group 2, labeled “university-society external link,” was mainly represented by members of social organizations (*N* = 4), followed by other groups: staff from public institutions (*N* = 2), university expert personnel (*N* = 2), and research center staff (*N* = 2).

In addition to achieving broad representation from the educational community as well as relevant professional personnel, generational representativeness was sought, with participants’ ages ranging from 21 to 60 years. Regarding gender, although a more balanced sample would have been desirable, the two focus groups had a higher proportion of female participants than male participants. This imbalance may be due to a selection bias among students and academic staff, as individuals interested in participating in the focus groups were from the social sciences and humanities, fields that inherently exhibit such gender selection biases (20).

Finally, in terms of educational level, participants held qualifications ranging from high school diplomas to PhD degrees, covering a broad spectrum of educational attainment. For a detailed description of the sample characteristics, see Tables 1, 2.

**Table 1:** Participants of the focus group “educational community.”

Cod.	Role	Age	Gender	Educational level
A.1	Academics	54	Female	PhD
A.2	Academics	31	Female	PhD
A.3	Academics	60	Male	PhD
PAS.1	Permanent administrative staff	49	Female	High school
PAS. 2	Permanent administrative staff	60	Male	High school
RF.1	Research fellow	29	Female	PhD
S.1	Student	27	Female	High school
S.2	Student	24	Female	High school
S.3	Student	25	Male	High school
S.4	Student	21	Female	High school

**Table 2:** Participants of the focus group “university-society external link.”

Cod.	Role	Age	Gender	Educational level
EU.1	University expert	56 años	Female	PhD
EU.2	University expert	52 años	Female	PhD
NGO.1	Non-governmental organization	31 años	Male	Bachelor’s degree
NGO.2	Non-governmental organization	45 años	Male	Bachelor’s degree
NGO.3	Non-governmental organization	26 años	Male	Bachelor’s degree
NGO.4	Non-governmental organization	24 años	Male	Bachelor’s degree
PI.1	Public institution	42 años	Female	Bachelor’s degree
PI.2	Public institution	42 años	Female	Bachelor’s degree
RC.1	Research center	44 años	Female	PhD
RC.2	Research center	49 años	Female	PhD

### 2.2 Process

To achieve the objective outlined at the outset, the first step involved designing a common research protocol for all project partners. The protocol specifies the procedures to be followed for conducting the focus groups, as well as the key questions intended to guide the discussion within each group. In each focus group, there was a facilitator and an observer, both affiliated with a partner university. The facilitator was responsible for creating a safe environment in which participants could express themselves freely and have their opinions respected. The observer’s role was to collect information relevant to the project objectives and support the facilitator.

Two focus groups were to be formed, each comprising a minimum of five and a maximum of 10 participants. As a guideline, one focus group was to consist of representatives from the university educational community, while the other was to include a mix of university representatives and personnel from public administration and social organization related to the project’s thematic focus. Participant selection followed a purposive or judgmental sampling criterion (21), aimed at representing diverse members of the educational community as well as stakeholders engaged in collaboration with the university on the relevant topic.

Both sessions were conducted between November and December 2025 via the university’s Google Meet platform. The decision to hold the focus groups online was primarily due to the participation of faculty members based at different campuses across various cities in the province of Cádiz, making online meetings more time-efficient for both project staff and participants.

After explaining the project and research objectives, the facilitator informed participants about consent procedures. Following the electronic or digital signing of consent forms, the discussions commenced, guided by the facilitator’s questions. Both sessions were recorded and subsequently transcribed with participants’ permission.

### 2.3 Instruments for data collection and analysis

A semi-structured interview guide was used to direct the focus groups. This guide was created by one of the project partners and validated by the others, comprising a total of four main topics: (1) gender equality and respect for sex-gender diversity in the university; (2) educational presence of gender equality and sex-gender diversity in the university context; (3) review of existing training on gender equality and sex-gender diversity in the university; and (4) protocols and the situation of gender-based discrimination and LGBTQA+ phobia in the university. Each topic included three open-ended questions to guide the discussion.

The duration of the focus groups ranged from approximately 1 h and 45 min to 2.5 h. Once the sessions were transcribed, we move on to encoding it (A for Academics, PAS for Permanent Administrative Staff, S for Students, EU for University Experts, NGO for Non Governmental Organization, PI for Public Institutions and RC for Research Center). Once information was coded the analysis phase began with the support of NVIVO12. Specifically, a reflexive thematic analysis (22) with an interpretative approach was conducted to explore in depth the meanings underlying participants’ opinions and discourses. This approach allowed for the identification of elements that transcended the predetermined topics, constructing patterns or subcategories aimed at understanding the university’s role in promoting gender equality and sex-gender diversity. The content was analyzed from an active and situated researcher perspective, acknowledging the subjective, interpretative, contextual, and non-neutral nature of the knowledge produced.

### 2.4 Ethical considerations

The study was approved by the Accredited Scientific Ethics Committee of Pantheion University in Greece. Additionally, the ethical criteria proposed by Vázquez and Angulo (23) were adopted, including negotiation, collaboration, confidentiality, impartiality, equity, and commitment to knowledge. In line with these principles, as mentioned above, participants were provided with detailed information regarding the research objectives, and written informed consent was obtained from all adult participants. Finally, to safeguard the credibility of the findings, the results section includes verbatim quotations illustrating the analyzed categories. These excerpts were selected for their informational richness and representativeness of the discourses collected from the various educational actors, following a rigorous triangulation process.

## 3 Results

**Figure 1:**
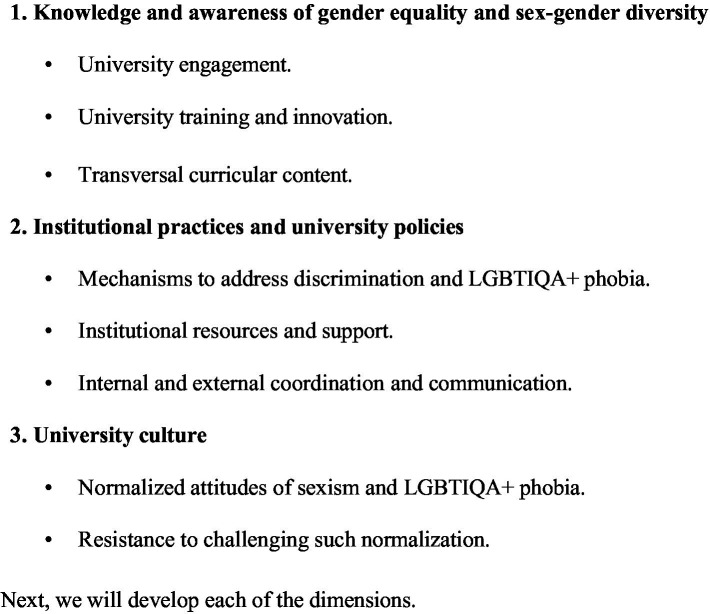
Summary of themes explored through the focus groups.

The results of this process were condensed into four dimensions (see Figure 1): (1) knowledge and awareness of gender equality and diversity (university engagement, training and innovation, and transversal curricular content); (2) institutional practices and university policies (mechanisms to address discrimination and LGBTIQA+ phobia, institutional resources and support, and internal and external coordination); and (3) university culture (normalized attitudes of sexism and LGBTIQA+ phobia and resistance to challenging such normalization).

### 3.1 Dimension 1: knowledge and awareness of gender equality and sex-gender diversity

This dimension focuses on three aspects: participants’ perceptions regarding university engagement, training and innovation, and the transversal integration of gender equality and sex-gender diversity into the curriculum.

Overall, regarding institutional engagement, both focus groups acknowledged the existence of protocols and equality units but perceived their applicability as limited: *“I would say there is a general commitment but…eh…there is a general lack of application…”* (PAS1). From participants’ everyday experience, institutional involvement is seen as more symbolic than effective (PAS2 and A1).

This perception extends to university training and innovation. Participants are aware of training initiatives on gender and diversity but consider them *“isolated or sporadic practices”* (A3, PAS1, S3), minimally innovative (A2, RF1), and often attended by students already sensitized (UE1) or motivated primarily by certificates (NGO1), or who do not perceive the topic as relevant due to a lack of observed homophobia (NGO3).

Behind these statements, participants recognize the need for more attractive and sustainable training programs, ideally with real incentives to foster participation across the entire university community, not just those already sensitized. Some participants associate such innovation with financial resources (UE2), while others suggest providing students with materials in diverse formats, preferably audiovisual and short in duration, to match digital demands (NGO4, RC2).

Several participants distinguish between training on gender equality and sex-gender diversity, perceiving the former as more prevalent and the latter as insufficient (PI2, NGO3). Despite these shortcomings, participants agree that even limited, isolated, and minimally innovative courses are crucial for the university community (UE2, A2, NGO1, S3). As one participant emphasized, these courses not only inform but also help prevent entrenched violent gender patterns (NGO4): *“What I would highlight regarding these courses is the possibility they represent to really mean something (…) the possibility to change our institution toward a more inclusive and feminist University”* (A1). Participants also noted a lack of transversal integration in the curriculum; content on gender equality and sex-gender diversity depends heavily on the individual teacher’s willingness (S1, PI1). Teaching or sensitizing students on these topics sometimes becomes a personal struggle: *“I have always been studying about gender equality and diversity, mainly because it touches me personally, but also because I believe diversity”* (NGO1). The absence of curricular integration, especially in teacher training, is inconsistent with the extensive regulatory framework (RC1): *“Although there is information, these things need to be addressed in the classroom and it is important that teachers are involved”* (NGO4).

### 3.2 Dimension 2: institutional practices and university policies

This dimension analyzes the mechanisms for responding to discrimination and LGBTIQA+ phobia, institutional resources and support, and internal and external coordination and communication. Regarding response mechanisms, participants acknowledged their existence, particularly protocols, but considered them inadequately applied, poorly understood (A2, NGO4), or excessively bureaucratic, discouraging their use: *“We have been working on the protocol for a while…but…but sometimes…there are so many issues to tackle…there is a lot of paperwork…sometimes it is not useful for the victims…and although they are meant to help…they could easily discourage victims to report”* (A1). This situation generates frustration: *“you do not feel comfortable at all with your academic institution”* (PAS2).

Another reason these mechanisms are underutilized is the normalization of discriminatory behaviors; certain comments and attitudes are considered ordinary, visible only when viewed through a “violet lens” (PAS1). Hate speech is also normalized among students, particularly through social media (NGO1), and reporting such incidents carries social consequences (RF1). Regarding institutional resources and support, participants generally perceived a lack of budget and human resources to implement measures, which affects program sustainability (S3). Coordination and communication in implementing and disseminating gender equality and diversity initiatives were also problematic. Participants noted poor communication between departments and faculties (A3, NGO3), sometimes due to underutilization of student communication channels (RC2). Students reported limited access to information, usually mediated by professors who conduct sporadic activities, often incentivizing attendance with certificates or class exemptions (S1, S2, PI2). Finally, participants emphasized that sex-gender diversity should be a priority in university policies to avoid being treated as a *“second-class issue, if you know what I mean”* (PAS1).

### 3.3 Dimension 3: university culture

University culture encompasses the ambivalences of the academic space, where institutional advancements (protocols, training courses, equality and diversity units) coexist with normalized practices, unenforced protocols, and a lack of genuine commitment. The university is thus a non-neutral space where power relations shape identity construction.

Two main aspects were analyzed: first, attitudes and discourses reproducing sexist and LGBTIQA+ phobic structures; second, spaces of resistance, particularly among sensitized faculty and students.

Regarding normalized attitudes and discourses, sexism and LGBTQA+ phobia are perceived as historically persistent in higher education, while younger children show greater acceptance of diversity (PI1, A3). Participants reported that non-binary peers *“have been calm,”* reflecting the normalization of violent patterns. Students *“make fun of others because they are gay”* (S1), and professors may express sexist or LGBTIQA+ phobic comments without consequence (S2). Awareness of these issues is often limited: *“Sometimes you cannot report it because you do not see it! When you are in a sexist society… you are used to it”* (PAS1), and *“people in higher positions turn a blind eye”* (S3).

Despite these challenges, acts of resistance exist as micro-political commitments to gender equality and diversity. Resistance occurs in teaching practices (NGO1, A3, RF1, A1), curricular content (A2, RF1, NGO4), identity statements (*“I am a feminist since birth”* PI1), and ethical engagement: *“We need to develop empathy… because we are getting worse and worse and violent situations are occurring every day”* (RC2); *“We should do something to motivate students”* (NGO4).

## 4 Discussion

The results of this study align with observations by authors such as Gairín Sallán et al. (42) regarding educational organizations: some institutions “learn” more than others, demonstrating the capacity to modify their structures, processes, and relationships, while others remain more instrumental than emancipatory. The latter may implement programs and protocols on sex-gender diversity and gender equality, yet fail to achieve real or transformative change. Lombardo and Maier (24) note that although universities adopt equality discourses, a significant gap persists between policy design and actual implementation, generating perceptions of inefficacy or distrust. This aligns with González et al. (25), who highlight the tension between formal organizational structures and organizational culture, whose complex interaction reproduces and perpetuates discriminatory practices.

Universities, as socializing institutions, are not neutral; they reproduce cultural frameworks that legitimize certain ways of being while marginalizing others. Participants perceive training initiatives on gender and sex-gender diversity as isolated, poorly integrated, and minimally innovative. Bardisa (26) emphasizes the gap between the formal organization—documented in regulations, policies, and plans—and the real organization, which manifests in everyday practices. When educational institutions fail to articulate a cohesive educational project, training initiatives are relegated to individual efforts or isolated interventions, failing to transform organizational culture as a whole. González et al. (25) further note that relationships among faculty in universities tend toward “cellularism,” where each professor conducts educational activities in isolation, with limited awareness of others’ work and without effective organizational controls.

Another explanation for the gap between formal structures and university culture, according to Meyer and Rowan (27), is that educational institutions function as loosely coupled systems, with internal elements maintaining relative autonomy. González et al. (25) also argue that bureaucratic organizational cultures prioritize process management over meaningful learning, focusing on methodological innovation rather than reviewing structures, communication channels, participation mechanisms, power relations, and other elements that shape the educational culture. Consequently, despite policies, regulations, and units dedicated to gender equality and sex-gender diversity, universities often fail to translate these formal commitments into everyday practices, generating perceptions that initiatives are symbolic rather than transformative.

The predominance of bureaucratic, process-oriented cultures limits universities’ capacity to foster inclusive and equitable environments. Traditional cultural frameworks continue to legitimize cisheteronormativity while marginalizing dissenting sexualities. Faculty training and innovation programs, although valuable, are insufficient if they do not challenge entrenched practices, professional habits, and institutional logics that sustain inequalities. Limited spaces for dialogue, coordination, and collective participation further hinder transversal integration of gender equality and sex-gender diversity. Challenges also persist in incorporating these perspectives into curricula, as many disciplines still treat them as supplementary or irrelevant, reproducing partial views of knowledge (28). University education that does not prioritize these subjects contributes to perpetuating inequalities among future professionals.

In addition, the study’s findings align with broader research on the experiences of LGBTIQA+ individuals in academia. Larsen et al. (29) highlight in their web project that working conditions, self-censorship, and concerns about academic freedom significantly affect LGBTQIA+ faculty and staff, limiting their ability to engage fully in institutional life. Gregory and Matthews (30) emphasize that social policies and institutional climates play a critical role in shaping opportunities for queer individuals to “come out” and participate openly, demonstrating that organizational culture and policy are deeply intertwined with lived experiences. Musubika (28) further stresses that perceptions of safety are central to student engagement in diversity, equity, and inclusion (DEI) initiatives, reinforcing the need for comprehensive institutional strategies rather than isolated interventions.

The concept of the queer university offers a critical horizon for addressing these challenges. As Ahmed (31) proposes, the contemporary university—traditionally seen as a space for knowledge production and legitimization—must rethink itself through epistemological frameworks that question cisheteronormative structures. The queer university is not limited to dissident sexual identities; it represents a pedagogical project that interrogates cisheteronormativity, challenges educational systems, and fosters alternative ways of producing and inhabiting knowledge (32). It functions as a critical and utopian horizon, destabilizing disciplinary, bureaucratic, and heteronormative logics that have historically dominated academia (33).

A queer university envisions horizontal, affective, and collaborative academic communities that prioritize well-being. Education becomes a practice of freedom, linked to emancipation, questioning, and ongoing dialogue, grounded in care (34). Faculty-student relationships are transformed to emphasize reciprocity, reflexivity, and the integration of lived experience (35). This approach challenges traditional dichotomies between theory and practice, academic and community knowledge, and thought and body (36). Structural transformations are required, including democratization of governance, depatriarchalization and deracialization of curricula, non-precarious working conditions, horizontal and embodied pedagogical practices, and openness to experimental thought (43).

Furthermore, queer theoretical frameworks provide practical guidance for teaching and institutional practices. Velinov and Hamza-Orlinska (37) show that incorporating queer perspectives into global diversity, equity, and inclusion education enhances reflexivity and challenges normative assumptions, encouraging more inclusive pedagogical and organizational practices. The queer university also emphasizes safe and affirming spaces for all students, where bodies are not subjected to control or binary classification, fostering alternative forms of existence, expression, and learning (38). It is not a finished project but an ongoing, collective practice that rearticulates critique as imagination, discomfort as political resource, and vulnerability as the basis for inclusive academic communities (39).

Integrating the queer university perspective with the study’s findings highlights the need for universities to move beyond symbolic compliance toward structural, cultural, and pedagogical transformation. Only through such comprehensive approaches can gender equality and sex-gender diversity become central to the identity and functioning of higher education institutions, improving experiences for students, faculty, and staff alike.

## 5 Conclusion

Achieving genuine inclusion in universities requires more than implementing policies, programs, or isolated initiatives; it demands structural, cultural, and pedagogical transformation. The study reveals a persistent gap between formal commitments to gender equality and sex-gender diversity and the realities of everyday university life. Bureaucratic and process-oriented cultures, fragmented implementation, and limited spaces for dialogue hinder the integration of inclusive practices, reinforcing cisheteronormative norms and marginalizing dissenting sexualities.

The findings suggest that moving beyond symbolic compliance necessitates embedding inclusion into the very identity and functioning of higher education institutions. This involves revising decision-making processes, power dynamics, work organization, and communication channels, as well as promoting collective participation and collaborative pedagogical practices. Curricula must prioritize gender and sex-gender diversity across disciplines, and universities must create safe physical and symbolic spaces, including gender-neutral facilities and inclusive cultural and leadership opportunities for LGBTIQA+ students.

In line with the concept of the queer university, genuine transformation requires imagining higher education as a space that critically interrogates cisheteronormative structures, fosters horizontal and affective communities, and integrates care, reciprocity, and reflexivity into academic life. This approach emphasizes that inclusion is not a fixed outcome but an ongoing, collective practice of rethinking institutional logics, knowledge production, and social relations within the university. Ultimately, fostering inclusive universities depends on sustained institutional commitment, adequate resources, faculty and staff training, and deep cultural change. Only by embracing these comprehensive strategies can higher education institutions move from declarative statements of equality to meaningful, everyday practices that advance justice, equity, and well-being for all members of the academic community.

## Data Availability

The datasets presented in this article are not readily available due to ethical and privacy restrictions. Requests to access the datasets should be directed to macarena.machin@uca.es.
